# Antibacterial Activity and Kinetics of *Litsea cubeba* Oil on *Escherichia coli*

**DOI:** 10.1371/journal.pone.0110983

**Published:** 2014-11-05

**Authors:** Wen-Ru Li, Qing-Shan Shi, Qing Liang, Xiao-Bao Xie, Xiao-Mo Huang, Yi-Ben Chen

**Affiliations:** State Key Laboratory of Applied Microbiology Southern China, Guangdong Provincial Key Laboratory of Microbial Culture Collection and Application, Guangdong Institute of Microbiology, Guangzhou, China; CAS, China

## Abstract

Litsea cubeba oil is extracted from the fresh fruits of *Litsea cubeba* by distillation. In this study, its chemical constituents, antibacterial activity, kinetics and effects against *Escherichia coli* were studied. Its minimum inhibitory concentration (MIC) and minimum bactericidal concentration (MBC) were both 0.125% (v/v) by toxic food method. Moreover, the antibacterial kinetic curves indicated 0.0625% (v/v) of litsea cubeba oil was able to prolong the growth lag phase of *E. coli* cells to approximate 12 hours while 0.125% (v/v) of litsea cubeba oil was able to kill the cells completely. Furthermore, transmission electron microscope (TEM) observation showed most *E. coli* cells treated with 0.125% (v/v) of litsea cubeba oil were killed or destroyed severely within 2 hours. The litsea cubeba oil might penetrate and destroy the outer and inner membrane of *E. coli* cells. Thus many holes and gaps were observed on the damaged cells, which led to their death eventually. The antibacterial effects of litsea cubeba oil mainly attributed to the presence of aldehydes, which accounted for approximately 70% in its whole components analyzed by GC/MS. Based on the antimicrobial properties, litsea cubeba oil would have a broad application in the antimicrobial industry.

## Introduction

Essential oils are aromatic oily liquids obtained from some aromatic plant materials by distillation, squeeze, and solvent extraction [1]–[3]. Due to their efficient antifungal and antibacterial activity [4]–[7], essential oils have been widely used in cosmetic, sanitary, and agricultural and food industries [8]. For example, *Litsea cubeba*, an evergreen tree grown fast and widely in Southern China, Japan and Southeastern Asia, is a traditional Chinese medicine for curing inflammation, headache and intoxication [9], [10]. Litsea cubeba oil extracted from the fresh fruits of *Litsea cubeba* by distillation is a kind of clear oily liquid with pale yellow to yellow color and sour lemon-like flavor. The compositions and the yields of oils from different parts of *Litsea cubeba* were different [11]. Besides, the composition of essential oils from the plant can differ in harvesting seasons and geographical sources [12], [13]. Litsea cubeba oil is often used as a flavor enhancer in foods, cosmetics, cigarettes and as an antimicrobial or insecticides [14]–[16]. Wang & Liu [11] studied the minimum inhibitory concentrations (MICs) of the oils from different parts of *Litsea cubeba* on *Bacillus subtilis*, *Enterococcus faecalis*, *Escherichia coli*, *Monilia albicans*, *Pseudomonas aeruginosa* and *Staphylococcus aureus* and found the oils had moderate antimicrobial activity. Yang et al. [17] found the oil from *Litsea cubeba* had good fungicidal activity. However, knowledge on the antimicrobial kinetics and mechanism of essential oils was limited in nowadays. Therefore, it is necessary to further study their antimicrobial efficacy and mechanism for the widespread application of essential oils.

In this paper, the compositions of commercially available litsea cubeba oil were analyzed by GC-MS. *Escherichia coli* was chosen as the tested strain. The antibacterial kinetics and effects of litsea cubeba oil against *E. coli* were studied. Furthermore, the correlations between the antibacterial efficacy and its constituents were explored. Results showed the litsea cubeba oil had moderate antibacterial activity on *E. coli* and had dose antibacterial and rapid bactericidal effects. Its antibacterial effects mainly attributed to the presence of aldehydes which accounted for approximately 70% in the whole components.

## Materials and Methods

### Chemical reagents, microorganism, mediums, and cultivation

Litsea cubeba oil was purchased from Guangzhou Baihua Flavours and Fragrances Company Ltd (Guangzhou, China), which is pure with a density of 0.869 g/ml. The strain of *Escherichia coli* ATCC 8739 was purchased from American Type Culture Collection (ATCC) and conserved in our laboratory. The Mueller-Hinton (MH) medium was purchased from Guangdong Huankai Microbial Sci. and Tech. Co., Ltd (Guangzhou, China) which contained the following ingredients: 2.0 g beef extract, 1.5 g soluble starch, and 17.5 g acid hydrolysate of casein. The Murller-Hinton Agar (MHA) medium, used for aerobic culture of *E. coli* in 37°C, was prepared by adding 1.5% agar in the MH. All solvents and reagents were of analytical grade.

### GC-MS analyses of the litsea cubeba oil

The constituents of litsea cubeba oil were analyzed similarly to our previous study [18] using a Thermo Finnigan Trace GC ultra-gas chromatograph equipped with Finnigan Trace DSQ mass spectrometer. In short, the operating conditions were programmed from 60 to 220°C at 10°C/min and injection port temperature was from 70°C to 250°C. Compounds were tentatively identified by comparing the mass spectra with those of authentic samples in the NIST MS library.

### Antibacterial activity measurements

The antibacterial activity was measured by poisoned food technique as described in our previous study with slight modification [18]. The experiment concentrations (v/v) of litsea cubeba oil were 0 (as control), 0.0156%, 0.0313%, 0.0625%, 0.125%, and 0.25% respectively. The bacterial concentration of *E. coli* was determined by measuring optical density (OD) at 600 nm (0.1 of OD_600_ corresponding to 10^8^ CFU/ml). The quantity of *E. coli* cells was about 10^6^ CFU in every plate. The plates sealed by parafilm were incubated at 37°C in an incubator for 7 days. The minimum inhibitory concentration (MIC) and the minimum bactericidal concentration (MBC) of litsea cubeba oil against *E. coli* were determined as showing no visible growth until incubated for 1 and 7 days separately. Two separate experiments were carried out in triplicate.

### Antibacterial kinetics of the litsea cubeba oil against *E. coli* strain

The antibacterial kinetics of litsea cubeba oil against *E. coli* was determined, as described in our previous study [18], [19] with slight modification. The experimental concentrations (v/v) of litsea cubeba oil were 0 (as control), 0.0156%, 0.0313%, 0.0625%, and 0.125% respectively. The cell concentrations of *E. coli* were all 10^6^ CFU/mL. All the experimental groups were incubated at 37°C and shaken with 150 rpm in a water bath shaker. The antibacterial kinetic curves were made based on the absorbance of optical density at 600 nm (OD_600_) determined by a spectrophotometer (UV-5200 UV/VIS spectrophotometer, Shanghai metash instruments co., Ltd., Shanghai, China). Two separate experiments were carried out in triplicate.

### TEM observation on the morphological structure of *E. coli* treated with litsea cubeba oil

The experimental method for observing the morphological structure *E. coli* treated with litsea cubeba oil was the same as described in our previous study [18], [19]. The experimental concentrations (v/v) of litsea cubeba oil were 0 (as control), 0.125%, and 0.25% respectively. The cell concentrations were all 10^6^ CFU/mL. The cultures were incubated at 37°C and shaken with 150 rpm in a water bath shaker for 2 h. Then the cell cultures were sampled and prepared for transmission electron microscope observation (TEM, Hitachi H-7650). Two separate experiments were carried out in triplicate.

## Results and Discussion

**Figure 1:**
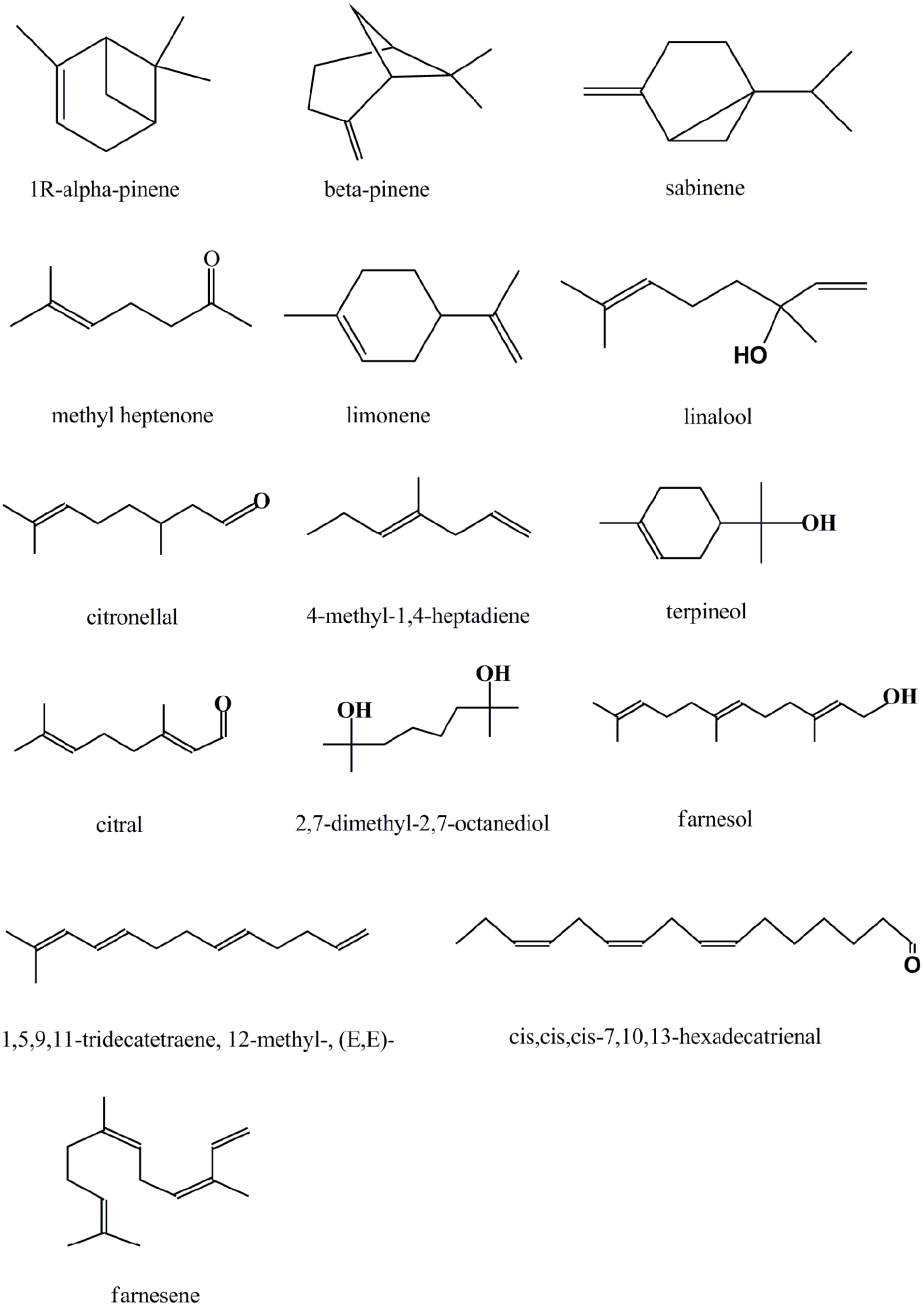
Chemical formula for constituents of litsea cubeba oil.

The main possible constituents of litsea cubeba oil identified by GC/MS are presented in Table 1 and the chemical formula is shown in Fig. 1. Results showed aldehydes (citral, citral isomer, citronellal, and citronellal isomer) were the main components of litsea cubeba oil, which accounted for approximately 70% in whole components. Alcohols (linalool, terpineol, 2,7-dimethyl-2,7-octanediol, and farnesol) accounted for approximately 4%. Alkenes (limonene, sabinene, 1R-α-pinene, 4-methyl-1,4-heptadiene, 1,5,9,11-tridecatetraene,12-methyl-,(E,E)-, cis,cis,cis-7,10,13-hexadecatrienal, β-pinene, and farnesene) accounted for approximately 23%. Antimicrobial activity of essential oils is closely related to their compositions, depending mainly on the functional groups. Previous study had been shown that the antimicrobial activity can be ranked from high to low as: phenols > cinnamic aldehyde > alcohols > aldehydes = ketones > ethers > hydrocarbons [20]. The antimicrobial activity of essential oil is mainly attributed to its major components though the minor constituents had a synergistic effect [12], [21]. Therefore, the antibacterial effects of litsea cubeba oil might mainly attribute to the presence of aldehydes, while other minor constituents such as alcohols, methyl heptenone, and alkenes played a synergistic antibacterial effect.

**Table 1:** Chemical constituents of litsea cubeba oil analyzed by GC/MS.

No.	RT/min	Area%	Name
1	5.7	1.35	1R-α-pinene
2	5.99	0.35	β-pinene
3	6.46	2.22	sabinene
4	6.51	1.83	methyl heptenone
5	7.4	15.94	limonene
6	8.43	2.45	linalool
7	9.24	1.34	citronellal
8	9.65	1.12	4-methyl-1,4-heptadiene
9	9.98	0.82	terpineol
10	10.85	32.08	citral isomer
11	11.36	36.17	citral
12	12.02	0.49	2,7-dimethyl-2,7-octanediol
13	12.48	1.05	citronellal isomer
14	12.76	0.14	farnesol
15	13.23	0.97	1,5,9,11-tridecatetraene, 12-methyl-, (E,E)-
16	15.28	0.59	cis,cis,cis-7,10,13-hexadecatrienal
17	21.84	0.32	farnesene
18		0.77	others

**Figure 2:**
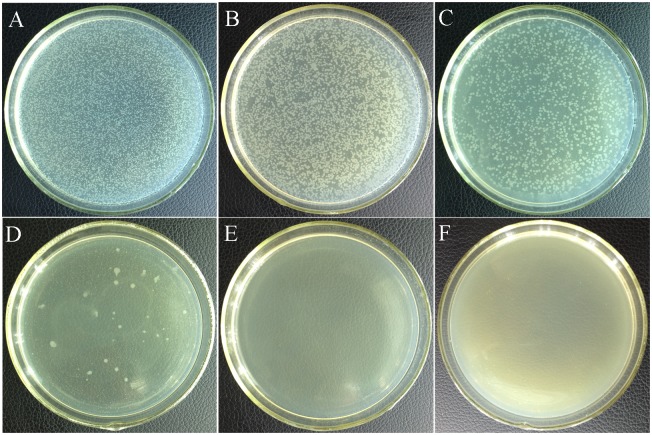
Experimental photos for determining antibacterial activity of litsea cubeba oil against *E. coli* treated for 1 day by poisoned food technique. (A) Control; (B) to (F) the experimental results with 0.0156%, 0.0313%, 0.0625%, 0.125% and 0.25% (v/v) litsea cubeba oil treatment respectively.

The experimental results for antibacterial activity of litsea cubeba oil determined by poisoned food technique were shown in Fig. 2. After incubation for 1 day, there were full of *E. coli* colonies in the Petri dishes of 0, 0.0156% and 0.0313% (v/v) experimental oil groups. But the quantity of *E. coli* colonies decreased with the increase of litsea cubeba oil concentration. When its concentration reached to 0.0625% (v/v), there were only dozens of colonies. Moreover, no any colony was observed in the Petri dishes of 0.125% and 0.25% (v/v) experimental oil groups. After incubation for 7 days, none bacteria colony was observed in 0.125% and 0.25% experimental oil groups yet. Therefore, its MIC and MBC against *E. coli* were both 0.125% (v/v) by the poisoned food technique. Compared with cassia oil whose MIC against *E. coli* was 0.025% (v/v) and main constituent was cinnamaldehyde [22], the litsea cubeba oil had moderate antibacterial activity. This is consistent with the antimicrobial activity of functional groups analyzed above. The experimental results also indicated litsea cubeba oil had a dose antibacterial effect.

**Figure 3:**
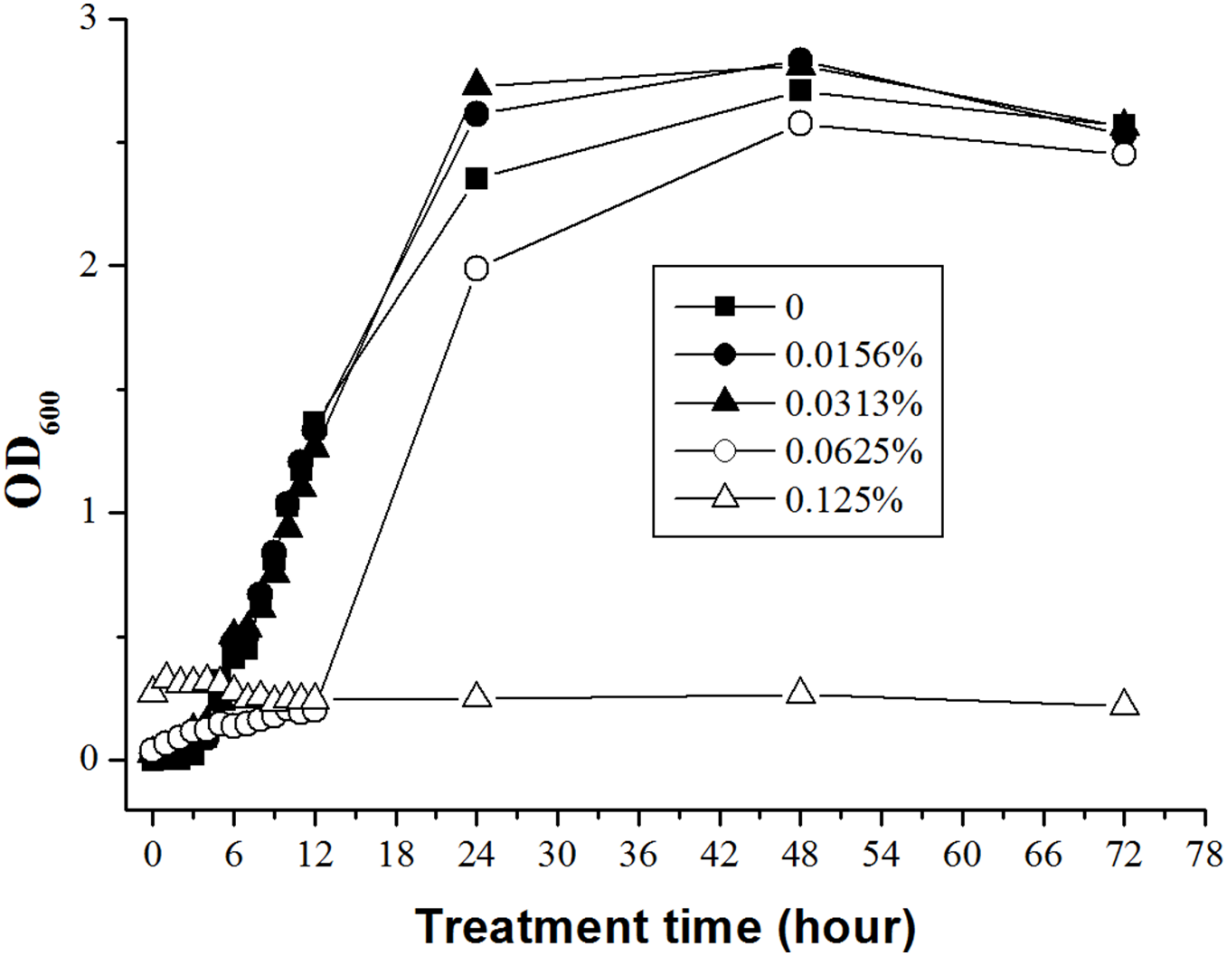
Antibacterial kinetic curves of litsea cubeba oil against 10^6^ CFU/mL *E. coli* cells. The concentrations of litsea cubeba oil were 0, 0.0156%, 0.0313%, 0.0625% and 0.125% (v/v) respectively.

The antibacterial kinetic curves of litsea cubeba oil against *E. coli* were shown in Fig. 3. The control group showed a typical growth curve including lag phase, exponential phase, and stabilization phase. The decline phase is not obvious because the OD_600_ value represented the total number of live and dead bacteria. The 0.0625% (v/v) of litsea cubeba oil could prolong the lag phase to approximately 12 hours, while the 0.0156% (v/v) and 0.0313% (v/v) experimental oil group didn’t show any antibacterial effect. Compare to Fig. 2 in agar medium, in which the 0.0156% and 0.0313% (v/v) experimental oil group showed weak antibacterial effect, the same experimental oil group in Fig. 3 in liquid medium didn’t show the same antibacterial effect. The possible reasons were as follows: The growth curves based on the OD_600_ represented the quantity of both the live and dead cells. So the curves couldn’t show the decrease of live cells in earlier treatment time., The quantity of live cells might decrease at initial treatment stage, but the tolerant cells gradually adapted to the environmental pressure and grew into exponential growth phase because of the weak antibacterial effect of 0.0156% (v/v) and 0.0313% (v/v) experimental oil groups. So it looked like no antibacterial effect. Moreover, the absorbance values of 0.0156% and 0.0313% (v/v) experimental oil groups were raised a little due to the absorbance of essential oils. The prolonged lag phase in 0.0625% (v/v) experimental oil group might imply a significant decline in the numbers of live cells in the initial treatment stage. So the small number of resistant cells spent more time to adapt to the environmental pressure and grow into exponential growth phase. When the oil concentration was up to 0.125% (v/v) no signs of growth were found within 72 hours in the growth curve. No live cells were determined in the samples from the 0.125% (v/v) either, which were streaked over the surface of MHA medium by sterile glass rod and incubated for 2 days at 37°C. So the 10^6^ CFU/mL *E. coli* cells in 0.125% (v/v) experimental oil group were killed completely after 72 hours. The antibacterial activity of litsea cubeba oil showed in Fig. 2 and Fig. 3 was similar and the MBC were both 0.125% (v/v). Therefore, the results indicated the oil had moderate antibacterial activity and a dose-antibacterial effect. Fungi are more sensitive than bacteria to essential oils [23], [24]. Some essential oils have strong antifungal activity but weak antibacterial activity such as citronella oil [25] and garlic oil [26]. However, litsea cubeba oil has both moderate antifungal [11], [17] and antibacterial activity.

**Figure 4:**
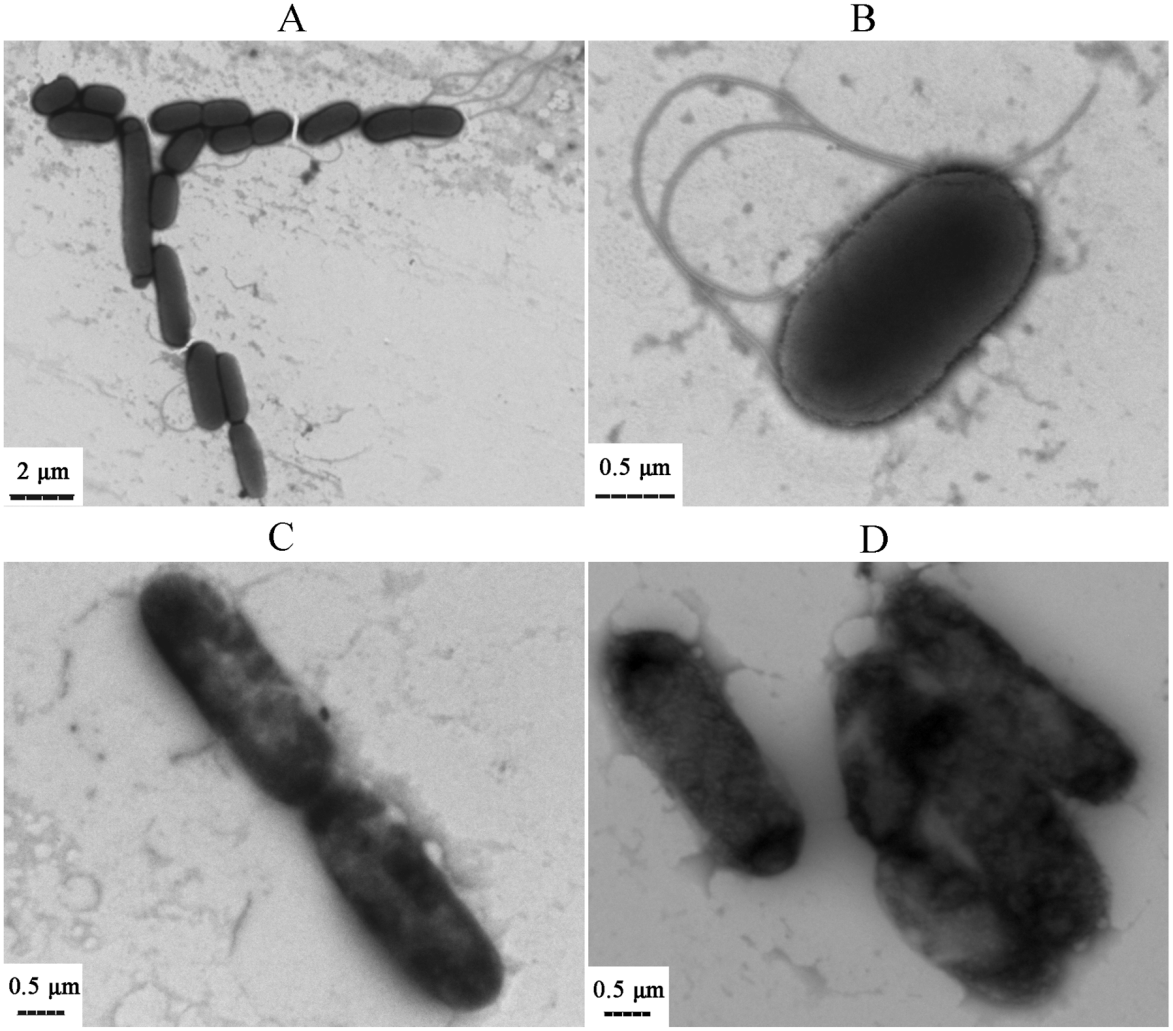
Photos for morphological alterations of *E. coli* cells observed by TEM. (A) to (B) Control; (C) 0.125% (v/v); (D) 0.25% (v/v).

The electron micrographs of *E. coli* cells were displayed in Fig. 4. The bacterial cells in control (Fig. 4a, b) showed typical characters of rod-shaped bacteria. The cell surface was smooth and intact, and the peritrichous flagella were obvious and clear. The cells treated with 0.125% (Fig. 4c) and 0.25% (v/v) (Fig. 4d) of oil for 2 h were destroyed severely. The flagella were damaged and even disappeared. This undoubtedly impeded the cells movement and accelerated their death. Moreover, bright and dark area appeared on the cell images, indicating the structure density of the cells varied. The bright area showed the cellular membrane was damaged. The cells in 0.25% (v/v) experimental oil group were destroyed more severely than that in 0.125% (v/v) experimental oil group. Essential oils can penetrate the plasma membrane because of their lipophilic characters [27], [28]. So the litsea cubeba oil might penetrate and destroy both outer and inner membrane of *E. coli* cells. Thus many bright holes and gaps were observed on the damaged cells and this led to their death. Then cell lysis happened and many cells were fragmentary. Moreover, the results also indicated litsea cubeba oil had a rapid bactericidal effect. Only treated with the oil for 2 h, the cells were both damaged severely and most cells were killed obviously. According to our experiments (data was not shown), the antibacterial effect of litsea cubeba oil was good but not long-lasting and its lemon aroma is strong but not long-lasting too. Its antibacterial property may be due to its rapid volatilization characters.

## Conclusion

In summary, the antibacterial activity of litsea cubeba oil might mainly attribute to the presence of aldehydes, which accounted for approximately 70% in the whole constituents. And other minor components play a synergistic antibacterial effect. Moreover, litsea cubeba oil had a moderate antibacterial activity. Its MIC and MBC were both 0.125% (v/v). Besides, it had dose-antibacterial and rapid bactericidal effects. Based on the antimicrobial properties, litsea cubeba oil would have a broad application prospect not only in the flavor spice industry, but also in the antimicrobial area.
